# DDIT3 switches osteogenic potential of BMP9 to lipogenic by attenuating Wnt/β-catenin signaling via up-regulating DKK1 in mesenchymal stem cells

**DOI:** 10.18632/aging.206091

**Published:** 2024-09-26

**Authors:** Hong-Hong Luo, Wen-Yan Ren, Ai-Hua Ye, Lu Liu, Yue Jiang, Fang-Lin Ye, Bai-Cheng He, Zhen-Hua Chen

**Affiliations:** 1 Key Laboratory of Biochemistry and Molecular Pharmacology of Chongqing, Chongqing Medical University, Chongqing 400016, China; 2 Department of Pharmacology, School of Pharmacy, Chongqing Medical University, Chongqing 400016, China; 3 Department of Pharmacy, Nanchong Central Hospital, The Second Clinical Medical School of North Sichuan Medical University, Sichuan 637100, China; 4 Department of Pharmacy, Nanchong Key Laboratory of Individualized Drug Therapy, North Sichuan Medical University, Sichuan 637100, China; 5 Department of Basic Medicine, Chongqing Nursing Vocational College, Chongqing 402763, China

**Keywords:** bone morphogenetic protein 9, DNA damage inducible transcript 3, osteogenesis, lipogenesis, Wnt/β-catenin

## Abstract

Bone morphogenetic protein 9 (BMP9) functions as a potent inducer of osteogenic differentiation in mesenchymal stem cells (MSCs), holding promise for bone tissue engineering. However, BMP9 also concurrently triggers lipogenic differentiation in MSCs, potentially compromising its osteogenic potential. In this study, we explored the role of DNA damage inducible transcript 3 (DDIT3) in regulating the balance between BMP9-induced osteogenic and lipogenic differentiation in MSCs. Utilizing techniques such as PCR, Western blot, histochemical staining, and *in vivo* experiments, we analyzed the osteogenic and lipogenic markers induced by BMP9 and delved into the underlying molecular mechanism. We found a significant upregulation of DDIT3 in C3H10T1/2 cells treated with BMP9. This upregulation led to a reduction in BMP9-induced osteogenic markers but an enhancement in lipogenic markers. Conversely, knocking down DDIT3 produced the opposite effects. Furthermore, BMP9-induced bone formation was decreased in the presence of DDIT3, but adipocyte formation was increased. Further investigations demonstrated that BMP9 increased the phosphorylation level of GSK-3β and promoted nuclear translocation of β-catenin, both of which were suppressed by DDIT3. Moreover, DDIT3 decreased the total β-catenin protein level while BMP9 increased the DKK1 protein level, which was further enhanced by DDIT3. Notably, knocking down DKK1 partially reversed the effect of DDIT3 on reducing BMP9-induced osteogenic markers and increasing lipogenic markers. Our findings indicated that DDIT3 enhances lipogenic differentiation by diminishing BMP9’s osteogenic potential, possibly through inhibiting Wnt/β-catenin signaling via DKK1 upregulation in MSCs.

## INTRODUCTION

Osteoporosis represents a highly prevalent orthopedic condition, particularly among postmenopausal women. Among the most efficacious treatment approaches is enhancing the osteogenic potential of bone marrow mesenchymal stem cells (BMSCs). Bone morphogenetic proteins (BMPs) belong to transforming growth factor β (TGF-β) superfamily. Several BMPs presented efficacious osteoblastic potential, such as BMP2, BMP7, and BMP9. Among these factors, BMP9 exhibits more stronger osteoblastic potential than any other BMPs, and it may also be a promising candidate for bone tissue engineering [1]. Whereas, there are several defects for BMP9 to induce bone formation, such as a long time for bone mature, and lipogenesis also occurred concomitantly [2]. Consequently, the BMP9 osteoblastic potential could be enhanced if these defects were effectively improved. It’s well-known that lipogenic and osteogenic lineage commitment are two mutually exclusive processes during the BMSCs fate decision. Namely, lipogenesis may be enhanced at the cost of osteogenesis, and vice versa. Thus, enhancing the osteogenic potential of BMP9 can be achieved through specific inhibition of adipogenesis.

The physiological functions of BMP9 can be mediated through classical or non-classical BMP/Smad pathways, such as PI3K/Akt and MAPKs [2, 3]. In addition, various other factors or signals are also involved in regulating BMP9-induced osteoblast differentiation, such as cyclooxygenase 2, insulin like growth factor 1, hypoxia inducible factor 1 alpha, Wnt/β-catenin, and Notch [1, 4–6]. So far, the specific mechanism by which BMP9 maintains the balance between osteogenic and lipogenic differentiation is not yet clear.

DNA damage inducible transcript 3 (DDIT3), a transcription factor with multiple functions triggered by endoplasmic reticulum stress, holds a pivotal role in not only modulating diverse cellular stress responses but also balancing osteogenic and lipogenic differentiation in mesenchymal stem cells (MSCs) [7–9]. However, the DDIT3 effect on osteoblastic or lipogenic differentiation remains a subject of ongoing debate and controversy. DDIT3, induced by endoplasmic reticulum stress, suppresses the differentiation of 3T3-L1 cells into lipogenic cells. Consequently, mice with DDIT3 knockout exhibited an increased accumulation of fat mass compared to wild-type mice [10], and bone formation was also diminished in DDIT3-null mice [11, 12]. In contrast, the osteogenic differentiation of MC3T3-E1 cells was enhanced by reducing the expression of DDIT3; furthermore, DDIT3 transgenic mice, driven by osteocalcin promoter, displayed a phenotype characterized by osteopenia [8]. Therefore, further research is necessary to delve deeper into the role of DDIT3 in regulating osteogenic and lipogenic differentiation.

In this research, we conducted both *in vitro* and *in vivo* experiments to uncover the potential role of DDIT3 in modulating the fate-determining capabilities of BMP9 in MSCs. Our findings could potentially pave a new way to enhance the osteogenic capacity of BMP9, thereby accelerating developments in regenerative medicine.

## MATERIALS AND METHODS

### Cell culture and chemicals

Cells were bought from American Type Culture Collection (USA). Mouse embryonic fibroblasts (MEFs) were extracted from NIH pregnant mice. Cells were cultured with Dulbecco’s modified Eagle’s medium containing 10% fetal bovine serum, 100 μg/ml streptomycin, and 100 U/ml penicillin; other conditions were set at 37°C and 5% CO_2_. Primary antibodies against Runx2 (sc-390715), OPN (sc-21742), β-catenin (sc-7963), GSK-3β (sc-377213), p-GSK-3β (sc-373800), DDIT3 (sc-7351), DKK1 (sc-374574), and C/EBPα (sc-365318) were all bought from Santa Cruz Biotechnology (China branch, Shanghai, China); primary antibody against GAPDH (10494-1-AP) was ordered from Proteintech (China branch, Wuhan, China).

### Recombinant adenovirus construction

The recombinant adenoviruses employed in this study were fabricated utilizing the AdEasy system [13]. Shortly, the mouse BMP9 and DDIT3 coding sequences were amplified with PCR. Then, the products, as well as the siRNA oligos of DDIT3 or DKK1 (sequences were shown in Table 1), were subcloned into shuttle vectors, respectively. Then, linearized and recombined with adenoviral backbone vector (pAdEasy1) in BJ/5183 bacteria. The products were linearized and transfected into HEK293 cells to package adenovirus. Recombinant adenoviruses were tagged with red fluorescent protein (RFP) or green fluorescent protein (GFP) for virus tracking, and the products were termed as AdBMP9, AdDDIT3, AdsiDDIT3, and AdsiDKK1. A recombinant adenovirus that solely expresses GFP (AdGFP) served as the control in this experiment.

**Table 1:** Sequence for siRNA in this study.

**Gene**	**GenBank entry**	**Oligo**	**Sequence (5ʹ→3ʹ)**
Dkk1	NM_010051.3	Sense 1	GCACCAAGCACAAACGGAA
		Antisense 1	UUCCGUUUGUGCUUGGUGC
		Sense 2	GAGGAAAGCAUCAUUGAAA
		Antisense 2	UUUCAAUGAUGCUUUCCUC
		Sense 3	ACAGAAAGAUCACCAUCAA
		Antisense 3	UUGAUGGUGAUCUUUCUGU
Ddit3	NM_007837.4	Sense 1	GCUCUCCAGAUUCCAGUCA
		Antisense 1	UGACUGGAAUCUGGAGAGC
		Sense 2	CCAGAUUCCAGUCAGAGUU
		Antisense 2	AACUCUGACUGGAAUCUGG
		Sense 3	GCUAGCUGAAGAGAACGAG
		Antisense 3	CUCGUUCUCUUCAGCUAGC

### RNA extraction and PCR assay

Total RNAs were extracted using the Trizol reagent, and their quality and concentration were quantified with a Nanodrop One instrument (Thermo Fisher Scientific, USA). Subsequently, 1 microgram of RNA was utilized to synthesize complementary DNA through a reverse transcription reaction employing a specific kit (No. R037A, Takara, Japan). CFX Connect system (Bio-Rad, USA) and SYBR green kit were used for real-time PCR assay. The program was set as follows: 95°C for 30 s; 95°C for 5 s, 60°C for 30 s, 40 cycles. Primers were listed in Table 2. The 2^−ΔΔCt^ method was used to calculate the relative mRNA expression level, and normalized with the mRNA level of glyceraldehyde triphosphate dehydrogenase (GAPDH).

**Table 2:** The primers used for PCR.

**Gene**	**GenBank entry**	**Primer**	**Sequence (5’ →3’)**
GAPDH	NM_008084	F	ACCCAGAAGACTGTGGATGG
		R	CACATTGGGGGTAGGAACAC
RUNX2	NM_001145920.1	F	GCCAATCCCTAAGTGTGGCT
		R	AACAGAGAGCGAGGGGGTAT
OPN	NM_001204233.1	F	TGCACCCAGATCCTATAGCC
		R	CTCCATCGTCATCATCATCG
DDIT3	NM_007837.4	F	AGTCCCTGCCTTTCACCTTG
		R	TCCGGAGAGACAGACAGGAG
C/EBPα	NM_007678.3	F	GCAGTGTGCACGTCTATGCT
		R	AGCCCACTTCATTTCATTGG

F, forward; R, reverse.

### Protein extraction and Western blot assay

After discarding the medium, the cells were washed twice with cold phosphate buffered saline (PBS, 4°C). Protein was extracted with radio immunoprecipitation assay lysis solution (RIPA; R0020-100, Solarbio, China) on ice. Lysates were centrifuged at 4°C (12000 g, 15 min). Supernatants were transferred to a new 1.5 ml EP tube, and boiled for 10 min. Proteins were separated using sodium dodecyl sulfate-polyacrylamide gels, and the gels were subsequently transferred onto a polyvinylidene fluoride membrane. After blocking with 5% bovine serum albumin for 1 h, membranes were incubated with primary antibody overnight at 4°C. Then, the horseradish peroxidase-labeled secondary antibody was used to blot the membrane for 1 h. Finally, proteins were developed with a chemiluminescent substrate kit (160072, Saimike Biotech, Chongqing China), and images were taken using ChemiScope 6200 gel imager (Qinxiang, Shanghai China). The data were quantified with Image J software.

### Alkaline phosphatase (ALP) staining

Cells were seeded in 24-well culture plates, and treated with AdGFP, AdBMP9, AdDDIT3, AdsiDDIT3, and AdBMP9 combined with AdDDIT3 or AdsiDDIT3. After treating for 5 or 7 days, ALP activities were measured by staining with kit (C3206, Beyotime, China). Afterward, the plates underwent scanning, and images were captured utilizing a microscope (IX53, Olympus, Japan) subsequently. The data were quantified with Image J software.

### Matrix mineralization assay

Cells were seeded in 24-well plates and treated with AdGFP, AdBMP9, AdDDIT3, AdsiDDIT3, and AdBMP9 combined with AdDDIT3 or AdsiDDIT3. After treating for 18 or 21 days, the medium was discarded and cells were gently washed twice with PBS (pH 4.2). Then, cells were fixed with 4% paraformaldehyde at room temperature for 20 min, and washed twice with PBS (pH 4.2). Finally, cells were stained with 0.4% Alizarin Red S solution [14]. Plates were scanned, and images were taken using a microscope (IX53, Olympus).

### Oil red O staining

Cells were seeded in 24-well plates and treated with AdGFP, AdBMP9, AdDDIT3, AdsiDDIT3, and AdBMP9 combined with AdDDIT3 or AdsiDDIT3. At the final stage, the medium was discarded, and the cells were rinsed twice with PBS. Subsequently, the cells were fixed with 4% paraformaldehyde at room temperature for 60 min. Following the fixation, cells were stained with a saturated Oil Red O solution for 30 min. After staining, the cells were washed twice again with PBS. The plates were then air-dried and scanned.

Finally, images of the cells were captured using a microscope (IX53, Olympus).

### Ectopic formation assay

The study involving animals was granted approval by the Animal Care and Use Committee at Chongqing Medical University (CQMU-2021-0197). The nude mice (female, aged 4-6 weeks, and weighing 20-24 g) were procured from Hua Fu Kang Biotechnology Company in Beijing, China. Cells were grown in 100 mm dishes and subjected to treatment with AdBMP9 or a combination of AdBMP9 and AdDDIT3. Following a 36-hour incubation period, cells were harvested and injected subcutaneously into the flank of the nude mice. After 4 weeks, all mice were euthanized, and the bone masses were retrieved for further histochemical analysis.

### Immunofluorescent staining

Cells were planted onto confocal dishes and then subjected to treatment with AdGFP, AdBMP9, AdDDIT3, or a combination of AdBMP9 and AdDDIT3. Following a 24-hour incubation, the cells were rinsed with PBS and fixed using 4% paraformaldehyde for 20 min. Subsequently, cells were washed with PBS and permeabilized with 0.5% triton X-100 for 20 min, following which they were rinsed twice with PBS. Next, the cells were blocked with 5% goat serum for 30 min and incubated overnight at 4°C with the primary β-catenin antibody. Following another double wash with PBS, the cells were stained with a Dylight594-conjugated secondary antibody. Finally, the cells were stained with diamidino phenyl indole for 10 min, rinsed twice with PBS, and imaged using confocal microscopy (SP8, Leica, Germany).

### Histological evaluation

The retrieved bone masses underwent fixation in 10% formalin, followed by a 10-day decalcification process using ethylenediamine tetraacetic acid decalcification solution. The samples were then embedded in paraffin. Afterward, the sections were deparaffinized, rehydrated, and stained with either hematoxylin and eosin or Masson’s trichrome. Finally, the stained sections were examined and photographed using a microscope (Ci-L, Nikon, Japan).

### Statistical analysis

The presented data are expressed as mean values with their corresponding standard deviations, and the results have been replicated in three separate experiments. Statistical significance was determined using either a one-way ANOVA or a two-tailed Student’s t-test. A *P*-value of less than 0.05 was considered indicative of a statistically significant difference.

## RESULTS

### Effects of BMP9 on DDIT3 expression in C3H10T1/2 cells

**Figure 1:**
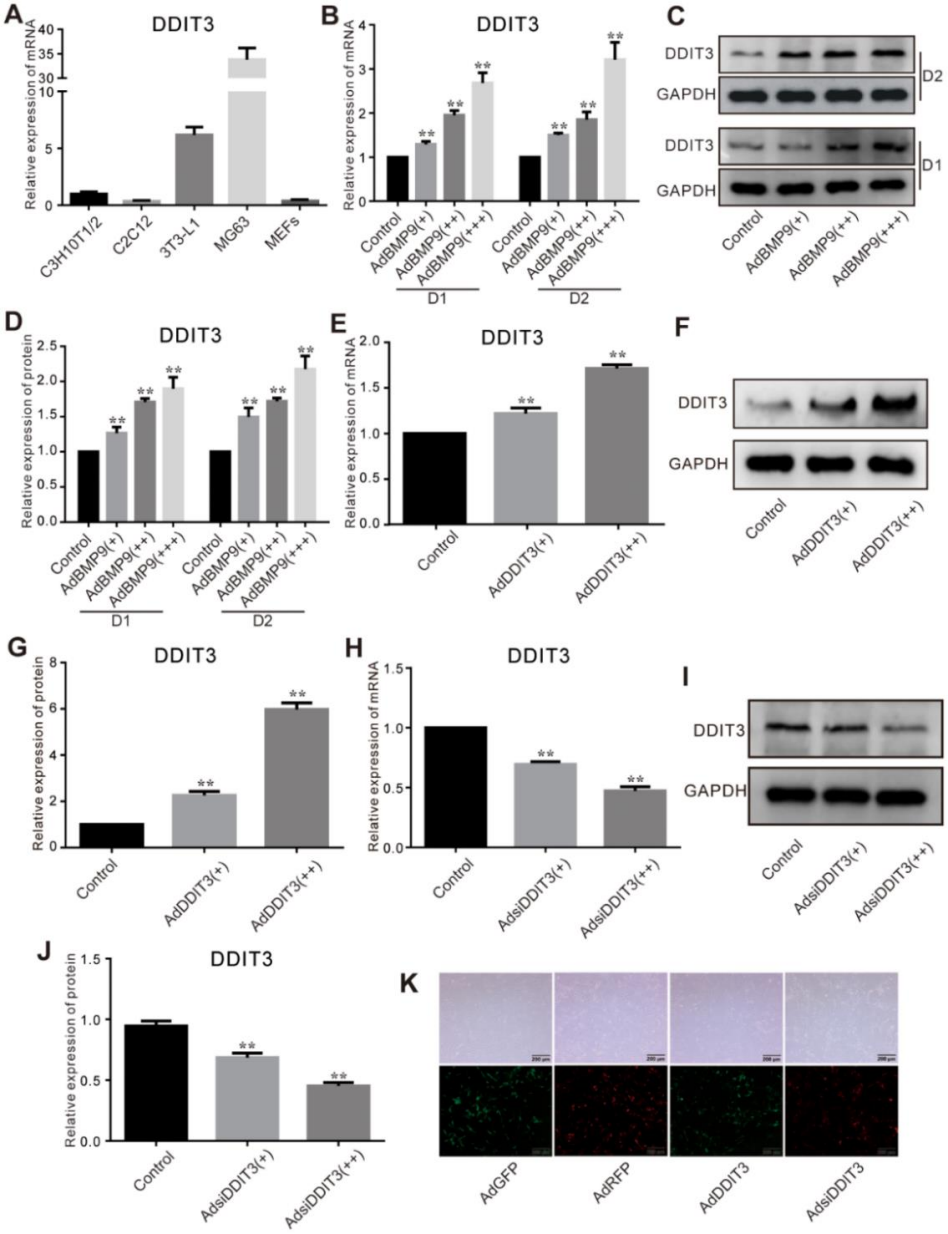
**Effects of BMP9 on the expression of DDIT3 in C3H10T1/2 cells.** (**A**) Real-time PCR assay shows the expression of DDIT3 in several progenitors and MG63 cells. (**B**) Real-time PCR assay shows the effect of BMP9 on the mRNA expression level of DDIT3. (**C**) Western blot assay shows the effect of BMP9 on the protein level of DDIT3. (**D**) Quantitative analyses of Western blot assay show the effect of BMP9 on the protein level of DDIT3. (**E**, **F**) Real-time PCR and Western blot assay show the effect of DDIT3 recombinant adenovirus on the mRNA and protein level of DDIT3. (**G**) Quantitative analyses of Western blot assay show the effect of DDIT3 recombinant adenovirus on the protein level of DDIT3. (**H**–**J**) Real-time PCR and Western blot assay show the effect of DDIT3 siRNAs recombinant adenovirus on the mRNA and protein level of DDIT3. (**K**) Images show the infection of AdDDIT3 and AdsiDDIT3 in C3H10T1/2 cells. “**” p <0.01; Compared with the BMP9 group. All results were repeated in three independent experiments.

DDIT3 mRNA was detectable in C3H10T1/2, C2C12, 3T3-L1, MEFs, and MG63 cells (Figure 1A). Given that C3H10T1/2 is a highly prevalent cell line utilized in lipogenic and osteogenic differentiation studies, it was selected for our subsequent research. Our findings revealed that BMP9 enhanced the expression of DDIT3 at both the mRNA and protein levels in C3H10T1/2 cells (Figure 1B–1D). The results indicated that DDIT3 is expressed in nearly all MSCs, suggesting a potential role of DDIT3 in modulating the osteogenic capacity of BMP9 in MSCs.

### DDIT3 effects on BMP9-induced osteogenic markers in C3H10T1/2 cells

**Figure 2:**
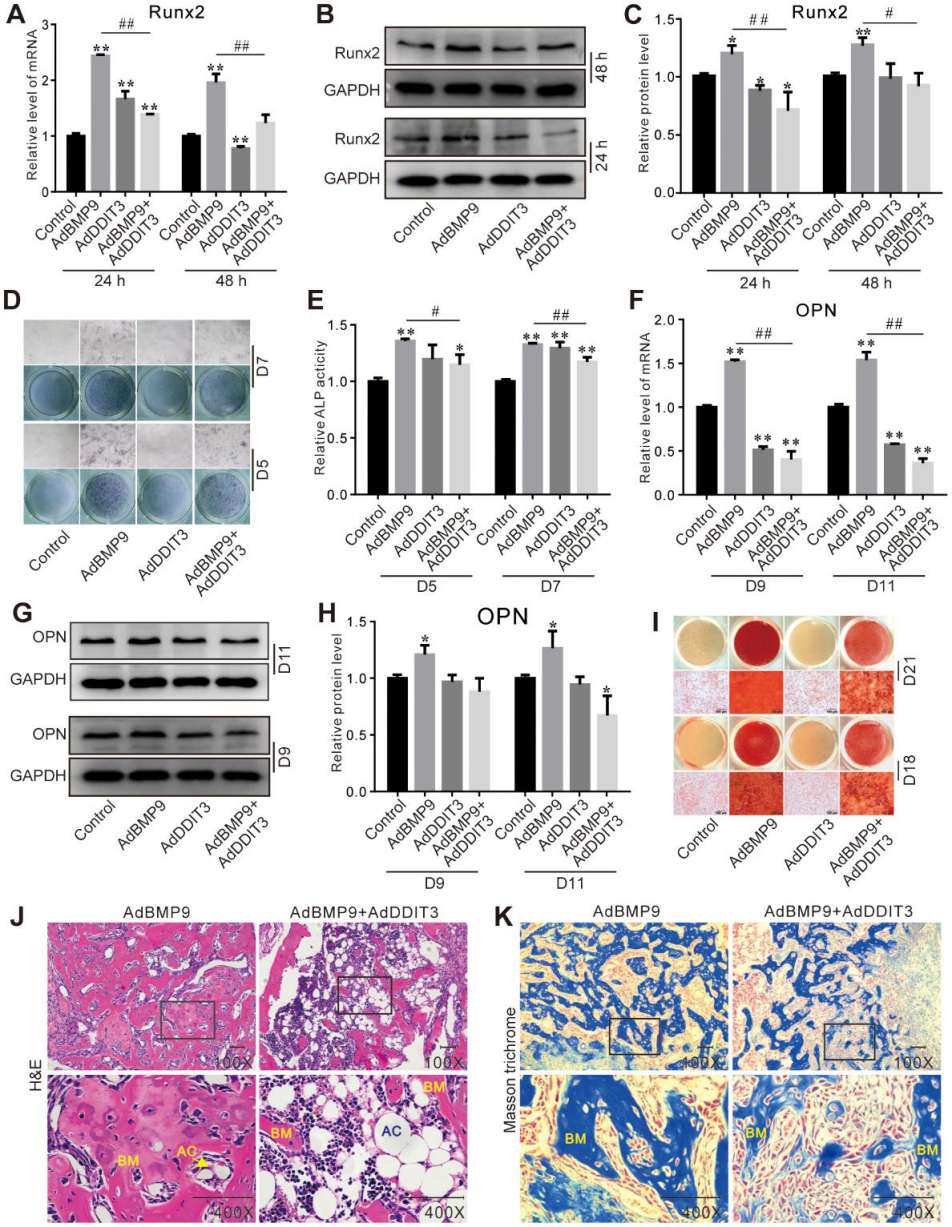
**Effects of DDIT3 on the BMP9-induced osteogenic markers in C3H10T1/2 cells.** (**A**) Real-time PCR results show the effect of DDIT3 on the mRNA expression of Runx2 induced by BMP9. (**B**) Western blot assay results show the effect of DDIT3 on protein level of Runx2 induced by BMP9. (**C**) Quantitative results of Western blot assay show the effect of DDIT3 on protein level of Runx2 induced by BMP9. (**D**) ALP staining results show the effect of DDIT3 on the ALP activity induced by BMP9 on day 5 or day 7. (**E**) Quantitative results of ALP staining show the effect of DDIT3 on the ALP activity induced by BMP9 on day 5 or day 7. (**F**) Real-time PCR results show the effect of DDIT3 on the mRNA expression of OPN induced by BMP9 on day 9 and day 11. (**G**) Western blot assay results show the effect of DDIT3 on the protein level of OPN induced by BMP9 on day 9 and day 11. (**H**) Quantitative results of Western blot assay show the effect of DDIT3 on the protein level of OPN induced by BMP9 on day 9 and day 11. (**I**) Alizarin red S staining results show the effect of DDIT3 on matrix mineralization induced by BMP9 on day 18 and day 21. (**J**) H&E staining results show the effect of DDIT3 on the bone formation induced by BMP9 (upper panel bar is 50 μm, lower panel bar is 200 μm; lower panel shows the detail about the part of the rectangle; BM: bone matrix, AC: adipocyte). (**K**) Masson‘s trichrome staining results show the effect of DDIT3 on the bone formation induced by BMP9 (upper panel bar is 50 μm, lower panel bar is 200 μm; lower panel shows the detail about the part of the rectangle; BM: bone matrix). Compared with the control group, “*” p <0.05, “**” p <0.01; compared with the BMP9 group, “#” p <0.05, “##” p<0.01. All results were repeated in three independent experiments.

BMP9 upregulated the expression of Runx2 mRNA and protein, which was subsequently partially downregulated by the overexpression of DDIT3 (Figure 2A–2C). On day 5 and 7, DDIT3 effectively suppressed the BMP9-induced ALP activities (Figure 2D, 2E). Similarly, on day 9 and 11, DDIT3 reduced the BMP9-induced OPN levels of mRNA and protein (Figure 2F–2H). Furthermore, DDIT3 significantly decreased the BMP9-induced matrix mineralization (Figure 2I). Additionally, the BMP9-induced bone formation was also attenuated by the overexpression of DDIT3 (Figure 2J, 2K). These results suggested that DDIT3 has the ability to diminish the osteogenic potential of BMP9 in C3H10T1/2 cells.

### Effects of DDIT3 knockdown on BMP9-induced osteogenic markers in C3H10T1/2 cells

**Figure 3:**
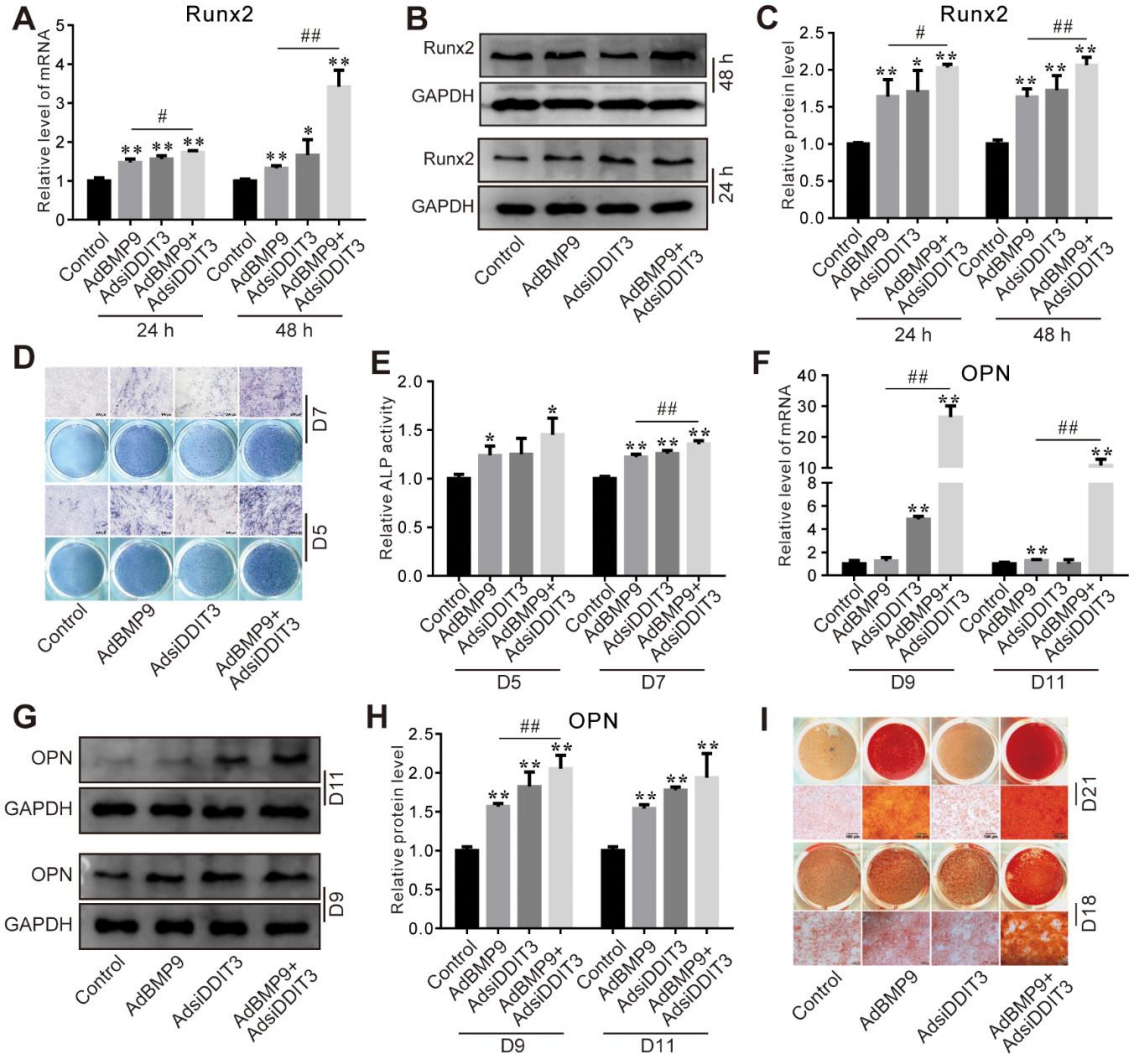
**Effects of DDIT3 knockdown on BMP9-induced osteogenic markers in C3H10T1/2 cells.** (**A**) Real-time PCR results show the effect of DDIT3 knockdown on the mRNA level of Runx2 induced by BMP9. (**B**) Western blot assay results show the effect of DDIT3 knockdown on the protein level of Runx2 induced by BMP9. (**C**) Quantitative results of Western blot show the effect of DDIT3 knockdown on the protein level of Runx2 induced by BMP9. (**D**) ALP staining results show the effect of DDIT3 knockdown on the ALP activity induced by BMP9 on day 5 or day 7. (**E**) Quantitative results of ALP staining show the effect of DDIT3 knockdown on the ALP activity induced by BMP9 on day 5 or day 7. (**F**) Real-time PCR results show the effect of DDIT3 knockdown on the mRNA expression of OPN induced by BMP9 on day 9 and day 11. (**G**) Western blot results show the effect DDIT3 knockdown on the protein level of OPN induced by BMP9 on day 9 and day 11. (**H**) Quantitative results of Western blot assay show the effect DDIT3 knockdown on the protein level of OPN induced by BMP9 on day 9 and day 11. (**I**) Alizarin red S staining results show the effect of DDIT3 on matrix mineralization induced by BMP9 on day 18 and day 21. “*” p <0.05, “**” p <0.01; compared with the BMP9 group, “#” p <0.05, “##” p<0.01. All data were repeated in three independent experiments.

Silencing of DDIT3 enhanced the stimulatory effect of BMP9 on the levels of RUNX2 mRNA and protein (Figure 3A–3C). Furthermore, the BMP9-induced ALP activities on day 5 and 7 were augmented by DDIT3 knockdown (Figure 3D, 3E), similarly to the BMP9-induced upregulation of OPN mRNA and protein levels on day 9 and 11 (Figure 3F–3H). Additionally, the BMP9-induced matrix mineralization was also strengthened on day 18 and 21 by DDIT3 silencing (Figure 3I). These results indicated that silencing DDIT3 expression can augment the osteogenic capabilities of BMP9.

### Effects of DDIT3 on the lipogenic markers induced by BMP9 in C3H10T1/2 cells

**Figure 4:**
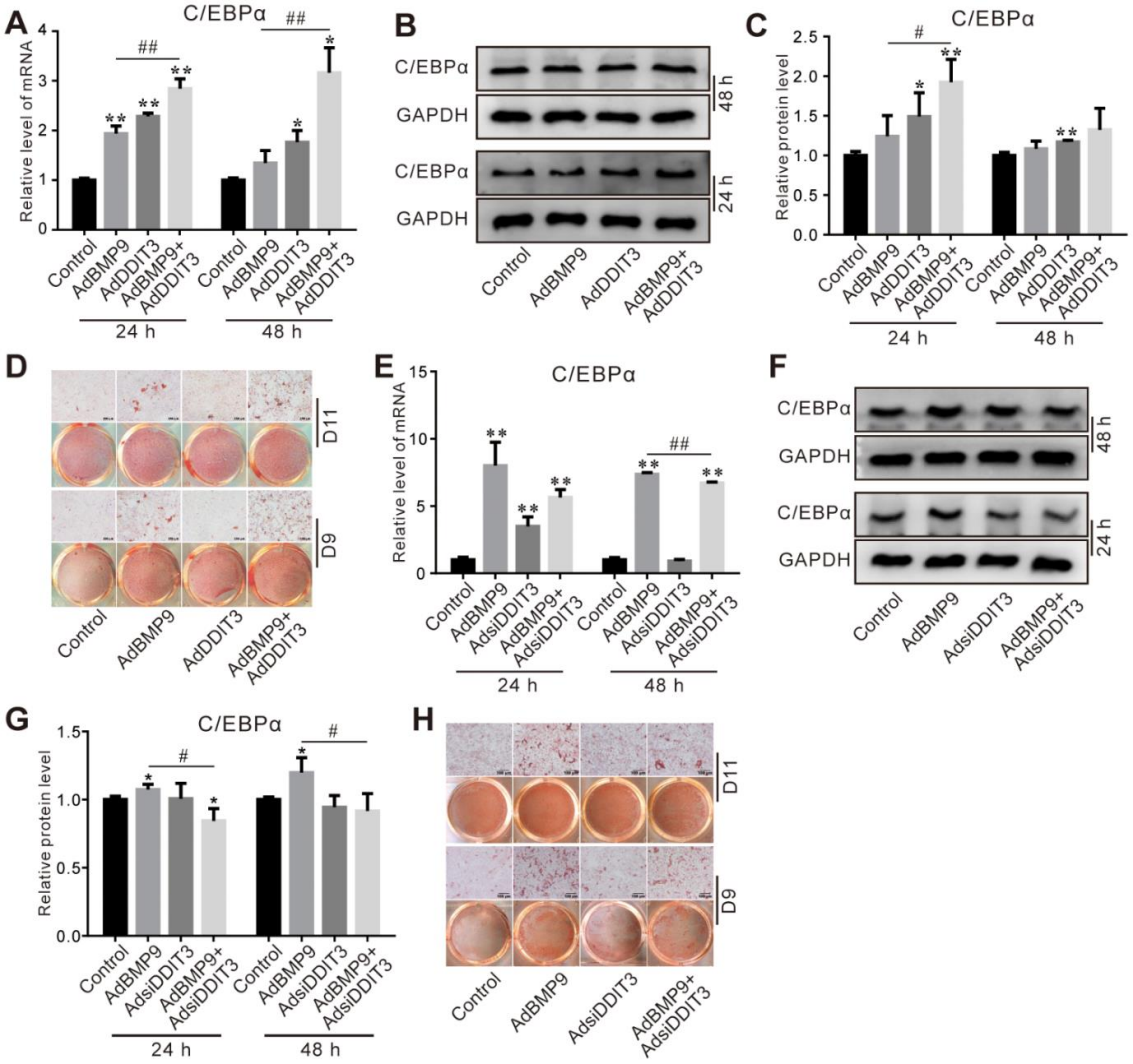
**Effects of DDIT3 on BMP9-induced adipogenic markers in C3H10T1/2 cells.** (**A**) Real-time PCR results show the effect of DDIT3 on the mRNA expression of C/EBPα induced by BMP9. (**B**) Western blot assay results show the effect of DDIT3 on the protein level of C/EBPα induced by BMP9. (**C**) Quantitative results of Western blot assay show the effect of DDIT3 on the protein level of C/EBPα induced by BMP9. (**D**) Oil red O staining results show the effect of DDIT3 on lipid droplet formation induced by BMP9 on day 9 and day 11. (**E**) Real-time PCR assay results show the effect of DDIT3 knockdown on the mRNA level of C/EBPα induced by BMP9. (**F**) Western blot assay results show the effect of DDIT3 knockdown on the protein level of C/EBPα induced by BMP9. (**G**) Quantitative results of Western blot assay show the effect of DDIT3 knockdown on the protein level of C/EBPα induced by BMP9. (**H**) Oil red O staining results show the effect of DDIT3 knockdown on lipid droplet formation induced by BMP9 on day 9 and day 11. Compared with the BMP9 group, “*” p <0.05, “**” p <0.01; “#” p <0.05, “##” p<0.01. All data were repeated at three independent experiments.

Next, we investigated the possible role of DDIT3 in modulating BMP9-induced lipogenic differentiation in C3H10T1/2 cells. The PCR and Western blot results revealed that the stimulatory effect of BMP9 on C/EBPα mRNA and protein levels was augmented by the overexpression of DDIT3 (Figure 4A–4C). Furthermore, DDIT3 also enhanced the BMP9-induced lipid droplet accumulation on day 9 and 11 (Figure 4D). Conversely, the BMP9-induced levels of C/EBPα mRNA and protein were reduced when DDIT3 was silenced (Figure 4E–4G), accompanied by a decrease in BMP9-induced lipid droplet accumulation on day 9 and 11 (Figure 4H). These results indicated that DDIT3 may enhance the lipogenic potential of BMP9 in C3H10T1/2 cells.

### Effects of DDIT3 and/or BMP9 on Wnt/β-catenin signaling in C3H10T1/2 cells

**Figure 5:**
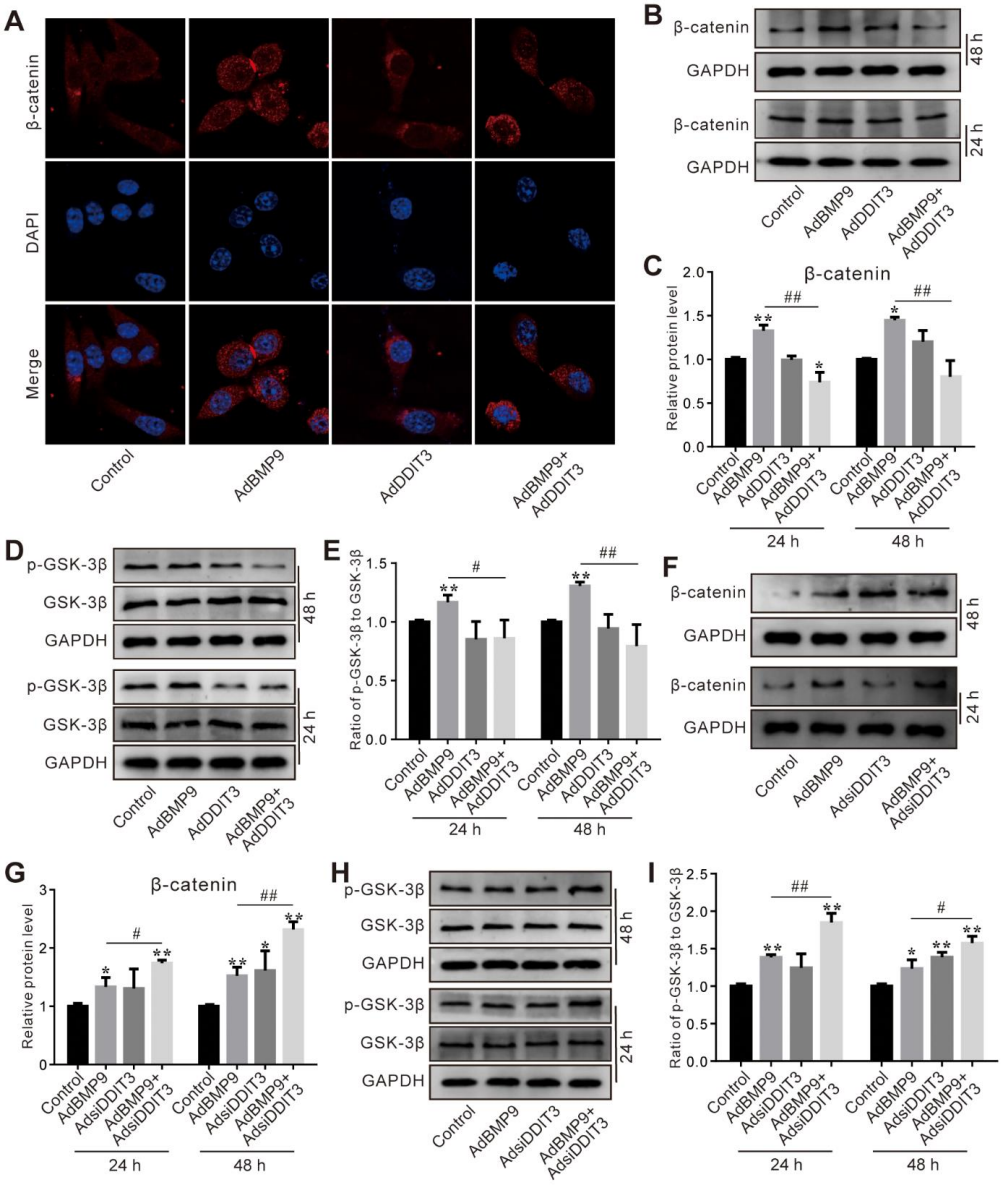
**Effects of DDIT3 on Wnt/β-catenin signaling affected by BMP9 in C3H10T1/2 cells.** (**A**) Confocal assay of immunofluorescent staining results shows the effect of DDIT3 on nucleus location of β-catenin induced by BMP9. (**B**) Western blot assay results show the effect of DDIT3 on the protein level of β-catenin affected by BMP9. (**C**) Quantitative results of Western blot assay show the effect of DDIT3 on the protein level of β-catenin affected by BMP9. (**D**) Western blot assay results show the effect of DDIT3 on phosphorylation of GSK-3β (p-GSK-3β) and total GSK-3β induced by BMP9. (**E**) Quantitative results of Western blot assay show the effect of DDIT3 knockdown on the ratio of p-GSK-3β to GSK-3β affected by BMP9. (**F**) Western blot assay results show the effect of DDIT3 knockdown on the level β-catenin affected by BMP9. (**G**) Quantitative results of Western blot assay show the effect of DDIT3 knockdown on the level β-catenin affected by BMP9. (**H**) Western blot assay results show the effect of DDIT3 knockdown on the protein level and phosphorylation of GSK-3β affected by BMP9. (**I**) Quantitative results of Western blot assay show the effect of DDIT3 knockdown on the ratio of p-GSK-3β to GSK-3β affected by BMP9. “*” p <0.05, “**” p <0.01, compared with the BMP9 group; “#” p<0.05, “##” p<0.01. All data were repeated in three independent experiments.

The immunofluorescent staining results showed that the BMP9-induced β-catenin nuclear accumulation was significantly reduced by DDIT3 (Figure 5A), which suggested that the effect of BMP9 on increasing the activity of Wnt/β-catenin may be attenuated by DDIT3. Further analyses of the results showed that the BMP9-increased total β-catenin protein level was also reduced by DDIT3 (Figure 5B, 5C). Similarly, the BMP9-induced GSK-3β phosphorylation was reduced by DDIT3, but no obvious effect on the total GSK-3β protein level (Figure 5D, 5E). Conversely, the BMP9 effect on increasing β-catenin protein level was enhanced by silencing DDIT3 (Figure 5F, 5G); the BMP9-induced phosphorylation of GSK-3β was increased by silencing DDIT3, although the total protein level of GSK-3β was unchanged (Figure 5H, 5I). These results indicated that the suppressive effect of DDIT3 on BMP9’s osteogenic potential may, in part, be due to its ability to reduce the activity of the Wnt/β-catenin signaling pathway.

### Effects of DDIT3 and/or BMP9 on DKK1 expression in C3H10T1/2 cells

**Figure 6:**
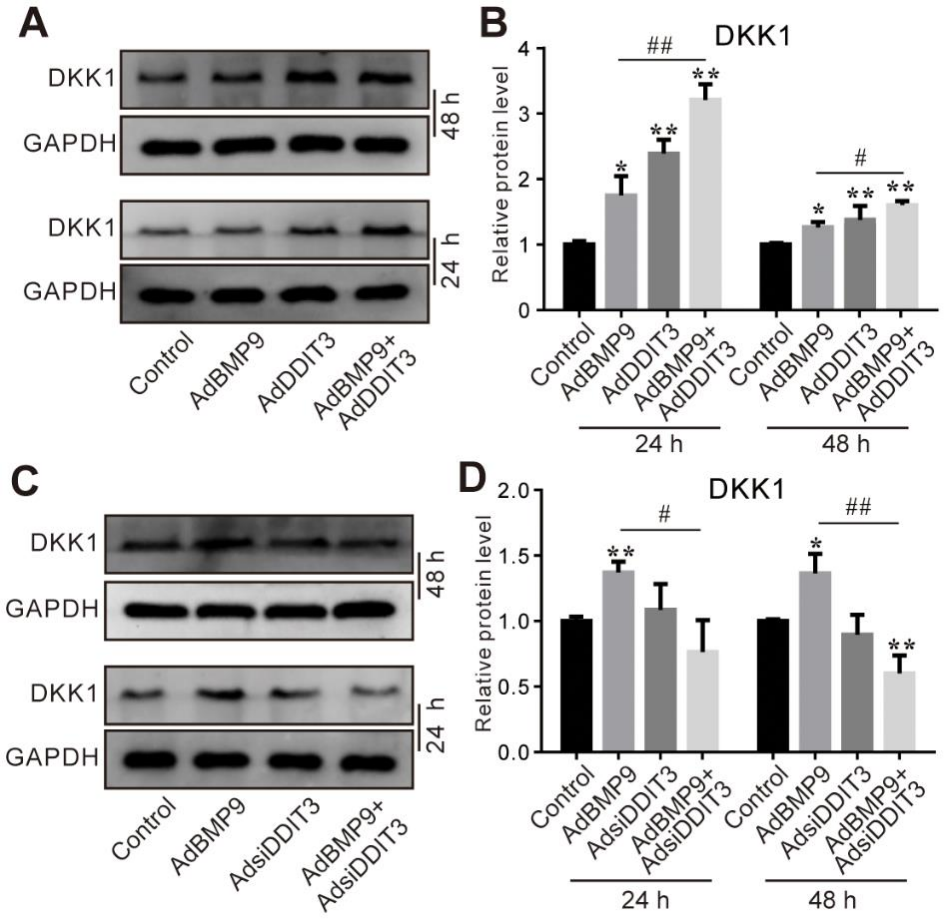
**Effects of DDIT3 and/or BMP9 on DKK1 expression in C3H10T1/2 cells.** (**A**) Western blot assay results show the DDIT3 effect on the protein level of DKK1 induced by BMP9. (**B**) Quantitative results of Western blot assay show the DDIT3 effect on the protein level of DKK1 induced by BMP9. (**C**) Western blot assay results show the protein level of DKK1 induced by BMP9 was reduced by DDIT3 knockdown. (**D**) Quantitative results of Western blot assay show the DDIT3 effect on DKK1 induced by BMP9. “*” *P* <0.05, “**” p <0.01, compared with the control group; “#” p <0.05, “##” p<0.01, compared with the BMP9 group. All data were repeated in three independent experiments.

Since DKK1 is a crucial negative modulator of the Wnt/β-catenin signaling pathway, we further investigated whether DDIT3 can suppress this pathway via DKK1. The Western blot assay results showed that the protein level of DKK1 was increased by BMP9 or DDIT3, and the BMP9 effect on increasing DKK1 protein level was enhanced by DDIT3 (Figure 6A, 6B). Contrarily, DKK1 protein levels were reduced when DDIT3 was silenced, and the BMP9 effect on increasing DKK1 protein level was inhibited greatly by silencing DDIT3 (Figure 6C, 6D). These results suggested that the attenuation of BMP9-triggered Wnt/β-catenin signaling activation by DDIT3 in C3H10T1/2 cells might be achieved through the upregulation of DKK1.

### Effects of DKK1 knockdown and/or DDIT3 on BMP9-induced osteogenic and lipogenic markers in C3H10T1/2 cells

**Figure 7:**
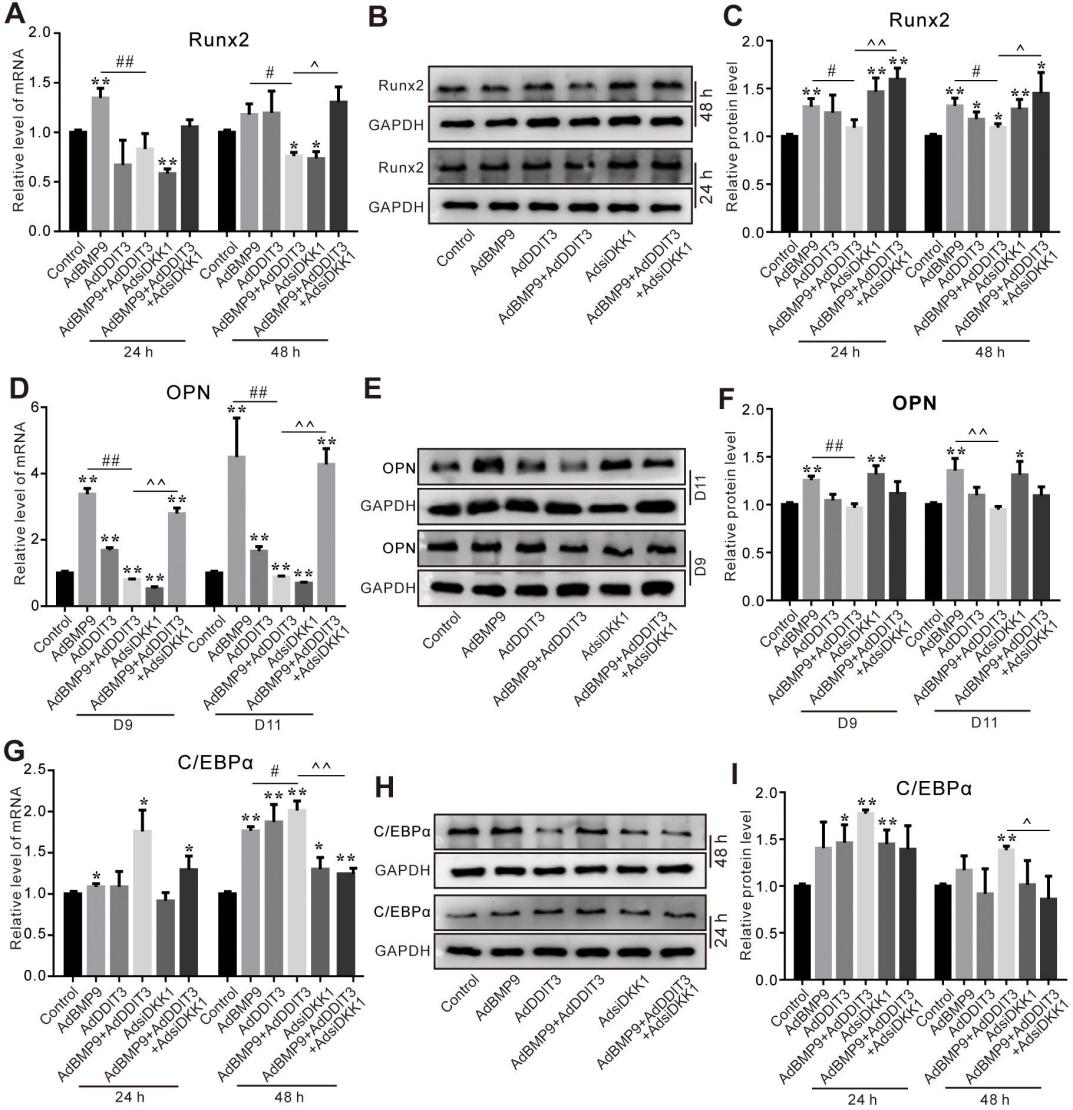
**Effects of DKK1 knockdown and/or DDIT3 on BMP9-induced osteogenic and adipogenic markers in C3H10T1/2 cells.** (**A**) Real-time PCR assay shows the effect of DDIT3 and/or DKK1 knockdown on Runx2 mRNA expression induced by BMP9. (**B**) Western blot results show the effect of DDIT3 and/or DKK1 knockdown on Runx2 protein level induced by BMP9. (**C**) Quantitative results of Western blot assay show the effect of DDIT3 and/or DKK1 knockdown on the Runx2 protein level induced by BMP9. (**D**) Real-time PCR assay shows the effect of DDIT3 and/or DKK1 knockdown on the mRNA expression of OPN induced by BMP9. (**E**) Western blot assay shows the effect of DDIT3 and/or DKK1 knockdown on the OPN protein level induced by BMP9. (**F**) Quantitative results of Western blot assay show the effect of DDIT3 and/or DKK1 knockdown on the OPN protein level induced by BMP9. (**G**) Real-time PCR assay results show the effect of DDIT3 and/or DKK1 knockdown on C/EBPα mRNA expression induced by BMP9. (**H**) Western blot assay shows the effect of DDIT3 and/or DKK1 knockdown on the C/EBPα protein level induced by BMP9. (**I**) Quantitative results of Western blot assay show the effect of DDIT3 and/or DKK1 knockdown on C/EBPα protein level induced by BMP9. “*” p <0.05, “**” p <0.01, compared with the control group; “#” p <0.05, “##” p<0.01, compared with the BMP9 group; “^” p <0.05, “^^” p<0.01, compared with DDIT3 and BMP9 group. All data were repeated in three independent experiments.

Finally, we investigated whether DKK1 plays a role in modulating the influence of DDIT3 on BMP9’s osteogenic capabilities. The PCR assay results showed that the inhibitory effect of DDIT3 on reducing BMP9-induced Runx2 mRNA expression was reversed by silencing DKK1 effectively (Figure 7A); similar results were found for the Runx2 protein level (Figure 7B, 7C). The DDIT3 inhibitory effect on BMP9-induced OPN mRNA and protein levels were almost restored by silencing DKK1 (Figure 7D–7F). In addition, the DDIT3 effect on increasing BMP9-induced C/EBPα mRNA and protein level was reduced apparently by silencing DKK1 (Figure 7G–7I). These results suggested that the effect of DDIT3 on modulating BMP9-induced osteogenic and lipogenic differentiation in C3H10T1/2 cells may, in part, be achieved through the upregulation of DKK1.

## DISCUSSION

While BMP9 possesses significant osteogenic properties, its osteogenic potential requires reinforcement due to certain limitations. Therefore, in this study, we delved into the role of DDIT3 in modulating BMP9’s osteogenic and lipogenic potential. Our study revealed that DDIT3 boosts BMP9’s lipogenic potential but comes at the cost of osteogenic differentiation, which may be achieved by suppressing Wnt/β-catenin signaling through DKK1 upregulation. Our findings indicated that specifically targeting DDIT3 inhibition could serve as a potential strategy to strengthen BMP9’s osteogenic potential.

An imbalance in the fate determination of bone marrow stromal cells could be a contributing factor to a range of diseases, including obesity and osteoporosis [15]. Several BMP members, such as BMP2 and BMP7, have been proven as effective osteogenic factors [16]. BMP9 shows more stronger osteoblastic potential than BMP2 or BMP7 [17]. It can enhance bone anabolic function and modulate bone remodeling processes in mice that have undergone ovariectomy [18], as well as induce osteogenic differentiation in rat dental sac stem cells [19]. The physiological functions of BMP9 are usually mediated through BMP/Smad signaling, as well as other non-canonical BMP/Smad pathways [20]. In the BMP/Smad signaling cascade, BMP9 initially binds to ALK1 and ALK2 receptors, triggering the phosphorylation of Smad1/5/8. This phosphorylated Smad1/5/8 then forms a complex with Smad4, and this complex ultimately translocates to the nucleus to regulate the expression of downstream target genes [21]. However, there are still a few weak-points for BMP9, such as lipogenesis also occurred [2]. It is a widely established fact that osteogenic and lipogenic lineages mutually exclude each other, and an imbalance between these two differentiation ways can potentially lead to some bone-related disorders, including osteoporosis [22]. Consequently, a promising approach to enhance BMP9’s osteogenic capabilities could involve specifically limiting its lipogenic potential.

The Wnt/β-catenin pathway has been established as a crucial regulator of MSCs' fate determination [23], and its decreased activity could potentially influence the lineage fate of bone marrow stromal stem cells, causing a shift from osteoblast to adipocyte [24]. It can be activated when the typical Wnt ligands bind to the receptor complex of frizzled and lipoprotein receptor-related protein 5/6 [25]; meanwhile, it can also be inhibited by various specific antagonists, such as Dickkopf-1 and sclerostin [26, 27]. Activation of Wnt/β-catenin can increase bone mineral density and cancellous bone volume [23]. The BMP9-induced osteogenic differentiation in mouse embryonic fibroblasts was reduced obviously by silencing β-catenin [28], but promoted by activating Wnt/β-catenin signaling [29]. Meanwhile, activation of Wnt/β-catenin signaling can inhibit lipogenic differentiation in MSCs [26, 30, 31]. Hence, a feasible approach to enhance BMP9’s osteoblastic potential while suppressing lipogenesis could be available by elevating the activity of Wnt/β-catenin signaling in MSCs.

In addition to the Wnt/β-catenin pathway, endoplasmic reticulum (ER) stress also significantly contributes to the regulation of MSCs’ fate decision [32–34]. DDIT3, a member of CCAAT/enhancer-binding protein (C/EBP) family, can be induced by ER stress [35]; it serves a crucial function in governing both apoptosis and differentiation processes [36]. The osteogenic markers were improved after being treated with 4-phenyl butyric acid (4-PBA, an ER stress inhibitor) [37]; the functionality of osteoblast was diminished, resulting in the development of osteopenia in DDIT3 transgenic mice [8]. On the contrary, the osteogenic capabilities of human dental pulp cells were augmented by overexpressing DDIT3 [38]. In addition, DDIT3 potentiated BMP4-triggered osteogenic differentiation while suppressing lipogenesis, likely via the activation of Wnt/β-catenin signaling [39]. DDIT3 potentially plays a role in the influence exerted by BMP2 in reversing the NR2F1-mediated suppression of osteoblast differentiation in MSCs [40]. The varying impacts of DDIT3 on the determination of MSCs’ fate could potentially contribute to the cell type and the microenvironment. Our results indicated that BMP9 upregulates DDIT3 expression in C3H10T1/2 cells. BMP9-induced osteogenic markers were suppressed by DDIT3 overexpression, while DDIT3 silencing exhibited a reversal effect. Conversely, DDIT3 enhances BMP9-induced lipogenic markers, which are suppressed by DDIT3 silencing. Our data indicated that DDIT3 may function as a crucial modulator, influencing the balance between osteogenic and lipogenic differentiation induced by BMP9 in MSCs.

Given that DDIT3 enhances BMP4-induced osteogenic differentiation via activating the Wnt/β-catenin signaling pathway [39], we hypothesized that the influence of DDIT3 on BMP9’s osteogenic and lipogenic potential may also be linked to this signaling. Our data showed that DDIT3 not only impedes β-catenin’s nuclear translocation but also diminishes the total protein level of β-catenin. Conversely, we observed the opposite effects when DDIT3 is silenced. Based on these results, we proposed that DDIT3 may diminish BMP9’s osteogenic potential by suppressing the activity of Wnt/β-catenin signaling pathway. DKK1, a soluble inhibitor of this signaling pathway, is crucial for embryonic head development [41]. By competitively binding to LRP5, DKK1 suppresses the Wnt/β-catenin signaling pathway, resulting in an augmentation of lipogenic differentiation in both rat tendon stem cells and human adipose-derived stem cells [25, 42, 43]. Previous studies have indicated that DKK1 impedes bone fracture repair, whereas suppressing DKK1 activity has been shown to promote bone formation in young animals [41, 44]. BMP9 enhances the expression of DKK1, yet its osteogenic capabilities are diminished by DKK1 overexpression [45]. In this study, we found that DDIT3 potentiated BMP9’s effect on upregulating DKK1, whereas silencing DDIT3 attenuated this effect. Additionally, the suppressive impact of DDIT3 on BMP9-induced osteogenic markers was significantly reversed when DKK1 was silenced. Conversely, DDIT3 promoted BMP9-induced lipogenic markers, which were suppressed by silencing DKK1.

In summary, our study revealed that DDIT3 has the capacity to diminish the osteogenic potential of BMP9 while enhancing its lipogenic potential. This effect of DDIT3 appears to be mediated through the upregulation of DKK1, leading to a reduction of Wnt/β-catenin signaling activity. The development of specific inhibitors or RNAi targeting DDIT3 may represent a promising approach to enhance BMP9’s osteogenic capability, potentially accelerating the progress of BMP9-based bone tissue engineering. However, further research is necessary to elucidate the precise mechanism underlying DDIT3-induced upregulation of DKK1.
